# Artificial cognition vs. artificial intelligence for next-generation autonomous robotic agents

**DOI:** 10.3389/fncom.2024.1349408

**Published:** 2024-03-22

**Authors:** Giulio Sandini, Alessandra Sciutti, Pietro Morasso

**Affiliations:** Italian Institute of Technology, Cognitive Architecture for Collaborative Technologies (CONTACT) and Robotics, Brain and Cognitive Sciences (RBCS) Research Units, Genoa, Italy

**Keywords:** embodied cognition, self-organization, body model, neural simulation of actions, developmental robotics, social robotics

## Abstract

The trend in industrial/service robotics is to develop robots that can cooperate with people, interacting with them in an autonomous, safe and purposive way. These are the fundamental elements characterizing the fourth and the fifth industrial revolutions (4IR, 5IR): the crucial innovation is the adoption of intelligent technologies that can allow the development of *cyber-physical systems*, similar if not superior to humans. The common wisdom is that intelligence might be provided by AI (Artificial Intelligence), a claim that is supported more by media coverage and commercial interests than by solid scientific evidence. AI is currently conceived in a quite broad sense, encompassing LLMs and a lot of other things, without any unifying principle, but self-motivating for the success in various areas. The current view of AI robotics mostly follows a purely disembodied approach that is consistent with the old-fashioned, Cartesian mind-body dualism, reflected in the software-hardware distinction inherent to the von Neumann computing architecture. The working hypothesis of this position paper is that the road to the next generation of autonomous robotic agents with cognitive capabilities requires a fully brain-inspired, embodied cognitive approach that avoids the trap of mind-body dualism and aims at the full integration of *Bodyware* and *Cogniware.* We name this approach Artificial Cognition (ACo) and ground it in Cognitive Neuroscience. It is specifically focused on proactive knowledge acquisition based on bidirectional human-robot interaction: the practical advantage is to enhance generalization and explainability. Moreover, we believe that a brain-inspired network of interactions is necessary for allowing humans to cooperate with artificial cognitive agents, building a growing level of personal trust and reciprocal accountability: this is clearly missing, although actively sought, in current AI. The ACo approach is a work in progress that can take advantage of a number of research threads, some of them antecedent the early attempts to define AI concepts and methods. In the rest of the paper we will consider some of the building blocks that need to be re-visited in a unitary framework: the principles of developmental robotics, the methods of action representation with prospection capabilities, and the crucial role of social interaction.

## 1 Introduction: artificial cognition vs. artificial intelligence

There is no doubt that AI (Artificial Intelligence) and ACo (Artificial Cognition) are frequently confused, in the engineering community as well as in the media, assuming that somehow are synonyms and functional for a common growing process of innovation in AI technologies. As a matter of fact, AI is a generic label that has been around since the 50’s of the last century, whereas the ACo label is quite recent, although the conceptual framework has been outlined at the same time of AI. Moreover, a similar confusion affects also the scientific community at large, including philosophy, psychology, and neuroscience: the issue is old and highly debated but still is far from allowing a shared definition of either Intelligence or Cognition, including the clear identification of similarities and differences. This also includes the use of quasi-oxymoronic expressions, as embodied intelligence, and is further clarified if we consider the etymology of intelligence and cognition.

Intelligence comes from the Latin verb *intelligere* and is defined as the Activity of the highest/purest part of the soul/mind (Latin “anima,” Greek 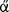
νεμοϛ or wind or breathing); more specifically, the etymology of Intelligence derives from the hierarchy of activities of living organisms defined by Aristotle, ordered from the lowest to the highest abstract layer: *vegetativum* → *sensitivum* → *motivum* → *intellectivum*. *Intelligere* applies only to *intellectivum*, implying that intelligence does not include perception and action but is only focused on the ideal forms or abstract essences of real phenomena: in summary, *intelligere* means to deal with impersonal, static knowledge, as far as possible from practical/bodily activities.

Cognition comes from the Latin verb *Cognoscere* and it is defined as the faculty of knowing, in the sense of the ability of an agent to learn and evaluate the surrounding reality. It is a personal, not impersonal faculty. Knowledge is not in the “cloud” but is acquired through personal experience in a dynamical way, filtered through the moving/sensing body as well as from past personal experience.

Thus, the main differences between intelligence and cognition for cooperative robots of the next generation can be summarized as follows:

• Cognition is embodied – Intelligence is likely to be dis-embodied.
• Cognition rejects the mind-body dualism – Intelligence implies the mind-body dualism.
• The goal of intelligence is to reason about encyclopedic knowledge and bounded by data.
• The goal of cognition is to improve the chance of personal survival, in the Darwinian as well as in the Enactivist sense, within a strong social context and exploiting internal models.

In summary, we suggest that Intelligence and Cognition correspond to two very different approaches to the acquisition, representation, accumulation, and exploitation of knowledge: Intelligence aims at abstract, impersonal, encyclopedic knowledge, independent of the personal and/or social process of action and interaction between brain, body, and environment; in contrast, Cognition deals with the individual process of knowledge acquisition through personal experience, possibly mediated by social interaction, a process constrained and guided by dynamics (between brain, body, and environment), development (ontogenetic as well as phylogenetic) and a value system of some type that is intrinsically alien to Intelligence *per se*. For example, *E* = *mc*^2^ is a piece of knowledge that captures the essence of a large variety of phenomena, independent of the humans that invented/discovered it and of the actual use by human society, e.g., understanding the nature of black holes vs. developing atomic bombs. This does not imply that Intelligence and Cognition are antithetic and mutually exclusive: on the contrary, they should be integrated carefully and “wisely,” in particular when we address the technological counterpart of such categories of knowledge, namely AI & ACo. On the other hand, it is not uncommon, in the neuroscience/cybernetic literature, to find expressions like “motor intelligence” that appear to contradict the distinction above and the crucial role of “embodiment.” In our opinion, this is just an example of the unavoidable ambiguity of natural language, including the language used in science: motor intelligence and related concepts are intrinsic embodied components of cognition and have little to share with disembodied, abstract intelligence. For AI a chair is a 3D shape while for ACo is a goal of the act of sitting.

At the same time, we should also consider a criticism of embodied cognition (Goldinger et al., 2016), suggesting that cognitive science (the whole of mental life, articulated in terms of perception, attention, memory, reasoning, and language) has little to gain from the posited integration of body and mind typical of embodied cognition. The main point is that, according to the authors, different forms of disembodied cognition, somehow in a similar way with the disembodied outline of AI, are characterized by a substantial underestimation of the fundamental concept of *action* in relation with other cognitive concepts as perception, attention, memory and so on. The cognitive importance of this concept is highlighted by the need of *prospection*, namely the mental simulation of actions for evaluating their potential sensorimotor effects in the future, either positive or negative, and thus supporting an informed decision-making process that escapes and bypasses the trap of trial and error. For example, mentally simulating “sitting” as part of the process to find a chair.

Historically, the starting point of the debate between AI and ACo can be identified with the invention of Cybernetics (Wiener, 1948), namely the discipline that deals with *Communication and Control in the Animal and the Machine*. Communication and Control are the basic ingredients when dealing with both Intelligence and Cognition and indeed Artificial Intelligence, including neural networks, is one of the many disciplines coming out from the cybernetic revolution. One of the hot issues that divided and still divides the cybernetic community at large is the degree of overlap between the application of cybernetic concepts to “Animals” or to “Machines”: in particular, to which extent engineers should focus on a bio or brain inspired approach for the design of intelligent/cognitive agents? Wiener himself was much in favor of a fundamental communality between natural and artificial prototypes/paradigms whereas the AI community was and is still oriented in the opposite direction, supporting the hypothesis that accelerating innovation methods of AI can approach a “different kind” of intelligence that, according to some “to be specified” metric, can even surpass human intelligence in many application areas, with a very thin degree of connection to biology and neuroscience.

The history of AI is marked by a number of booms and busts, driven by the dynamic interplay between science, technology, and economy. The last decade or so is clearly a booming period, marked by well publicized achievements in gaming (e.g., chess or go), computer vision and natural language technologies, driven by machine learning methodologies applied to deep neural networks. Artificial neural networks employed in AI are relatively simple and easy to use but are essentially “black boxes” whose connection weights can be refined through learning techniques, like back-propagation, without attempting any linkage with biology and neuroscience (another source of ambiguity of the term “neural networks”): the results are opaque for the designers and/or the user of the AI application, independent of the success of the app. In general, AI apps can make decisions or predictions based on parameters that the programmer has not defined and cannot deduce by looking at the output or the network code: the fundamental source of information behind the produced result is the intrinsic correlation hidden in the data used to learn. Two identical networks may behave in a quite different way depending on the random selection of the initial connection weights and, more importantly, on the choice of the big data set used for training the network as well as the order in which these data are acquired. This kind of opacity, due to the intrinsically passive nature of the learning mechanism, is clearly in contrast with standard engineering design methodologies related to safety and reliability and is likely to imply ethical and legal adoption problems as soon as the application domains include sensitive problems as medical diagnosis, autonomous driving, fraud detection, econometric analysis, and economic decision making. Moreover, the expansion of the application areas is motivating the growth of the number of layers and parameters of deep neural networks, supported by the continuous development of new software tools and the still increasing computational power: each layer of these networks represents the knowledge extracted from the training data at progressively more abstract levels, whose interpretation becomes more and more difficult to ascertain, highlighting fundamental limitations of the deep learning framework, such as catastrophic forgetting (French, 1999) and never-ending learning (Mitchell et al., 2018). Moreover, even in the specific application areas where AI technologies achieved unprecedented advancements, a strong impediment to the generally accepted adoption of such systems is “explainability,” namely the lack of transparency due to the black-box nature of these systems: they may provide powerful predictions that cannot be directly explained (Adadi and Berrada, 2018; Rahwan et al., 2019). The causal relationship between the acquired training data is lost and does not emerge.

Such problems motivated the rise of a recent discipline on a side of AI: Explainable Artificial Intelligence (XAI) (Barredo Arrieta et al., 2022). A major focus of XAI is not on the design and structural feature of such systems but on the audience for which explainability is sought and the different purposes of the explanations, e.g., explain to justify/control/improve performance/discover causal relationships. At the same time, the value of XAI is frequently called into question, claiming that the credibility of an AI system can be evaluated directly by monitoring the quality of the result over time and observing that modification of AI systems in such a way to explain every decision could decrease speed and efficiency. In any case, AI as well XAI research is far away from any brain-inspired formulation, somehow based on cognitive neuroscience.

Thus, it is somehow surprising that recent papers (Taylor and Taylor, 2021; Iglesias, 2022) associated Artificial Intelligence with Artificial Cognition^1^ proposing to “explain” the black boxes produced by AI technologies by means of the methods of analysis of the mind developed by Psychology and Cognitive Neuroscience, based on controlled stimuli and measurement of behaviour in various network architectures. The underlying rationale is that also the mind, investigated by such methods, can be treated as a black box and thus by importing cognitive methodology in the analysis of AI apps can allow to make causal inferences about the structure, architecture, and functioning of the black boxes generated by AI methods. This approach to XAI has been called **Artificial Cognition** and is based on the hope that experimental psychology can help generate explainable neural networks: the problem is that this is a post-hoc method that in no sense can be interpreted as a brain model.

In general, the definition of artificial cognition as a tool to measure decision making in artificial neural networks is clearly reductive, at best describing, not explaining, how the system works and does not consider a totally different approach for the development of cognitive architectures for autonomous agents that is fundamentally brain-inspired to start with and “proactive/exploratory” in nature. This approach can be traced back to the early times of AI although the name ACo was not specifically used and/or publicized but it is clearly behind the pioneering studies on Enactivism, Embodied Cognition and Self-Organization of living organisms (Maturana and Varela, 1980; Varela et al., 1991), leading to a formulation of cognitive neuroscience arguing that cognition arises through a dynamic interaction between an acting organism and its environment, mediated by a sensing and acting body, namely a purposeful, proactive, exploratory interaction that emphasizes the importance of adaptability and compositionality in artificial cognition while mirroring a fundamental organizational principle of the brain expressed as neural reuse: “…the brain as a dynamical system where individual regions are functionally diverse and are used and reused in many different tasks across the cognitive domain” (Anderson, 2010). In our opinion, this is the appropriate starting point for the development of reliable autonomous agents of the next generation, guided by the concept that acting is an aspect of decision making and perhaps the ultimate reason for a brain to exist.

In the following part of the paper, some crucial founding concepts and building blocks for conceiving and designing Artificial Cognitive agents will be outlined but we wish to emphasize that it is not *explainability per se*, as defined by XAI, the most urgent need for society, which demands to understand how and on what basis intelligent/cognitive agents support decisions that may affect everybody. One of the goals of AI is to achieve Artificial General Intelligence (AGI) that captures human cognitive abilities in general, aiming at a sort of “impersonal super-intelligence.” This would clearly overcome the understanding capability of any single individual, although there are reasons to doubt that this is an unrealistic and even impossible goal (Fjelland, 2020). We believe that a decision-making process based essentially on correlation, having lost any causal relationship between training data cannot “explain” the “why” of its outcome (Fjelland, 2020) but only “how” similar is the decision to past decisions. On the contrary, Cognitive Penetrability, in the sense defined by (Pylyshyn, 1999)^2^, allows humans to communicate and interact on the basis of a shared value system and this is the basis for achieving mutual trust in a cooperating society: for this reason, *cognitive penetrability* rather than *explainability* should be the starting point for the development of Autonomous Robotic Agents of the next generation.

In summary, the ACo formulation of the development of autonomous agents is fundamentally brain-inspired but its biomimetic approach is not intended to imitate the fully developed brain of Nobel prize winners or other remarkable cultural leaders but the process of progressive knowledge and competence acquisition, leading to autonomous decision-making ability. Thus, a fundamental building block of ACo robots can be outlined by re-visiting the basic issues of evolutionary robotics, emphasizing the difference between the data-driven, passive nature of training deep and large neural networks (the AI/AGI approach) and the active exploratory-driven knowledge acquisition nature of sensory-motor-cognitive development of human *cubs*. As suggested by von Hofsten (2004), “development is about systems with the urge to act and explore.” One crucial aspect of the developmental process is that it is made possible by internal and external constraints: the intrinsic plasticity of the brain and the persistent/sought interaction with the physical and social environment. The role and the computational mechanisms of the former type of interaction will be addressed in section 3 “Body model and Prospection capabilities for Artificial Cognition” and the latter type of interaction will be touched upon in section 4 “Social support of Developmental Artificial Cognition.” In particular, the issue of prospection will be linked to the memory system that accompanies and supports the accumulation of knowledge, at increasing levels of abstraction, on the basis of mental (covert) simulation of action and mental (covert) speech. Of course, this short list of items does not cover all the relevant components of ACo, such as the specific role of consciousness/awareness in artificial cognition and inner language in the development of cognition that are only briefly mentioned.

## 2 Principles of developmental robotics

In the context of artificial cognition, we suggest that a brain-inspired cognitive architecture for autonomous robotic agents of the next generation should consider general principles of Developmental Robotics. This is a multifaceted and interdisciplinary research area at the intersection of robotics, developmental psychology and developmental neuroscience. It is more than twenty years old (Sandini et al., 1998; Asada et al., 2001; Lungarella et al., 2003; Schmidhuber, 2006; Cangelosi et al., 2015; Min et al., 2016; Huang et al., 2017) and it clearly represents a brain-inspired approach to the design of robots: in contrast with industrial robots that perform repetitive predefined tasks in a predefined environment: these robots are supposed to be dynamic models of how humans develop, explore, and quickly adapt in an open-ended manner to a changing environment through lifelong learning, in order to cope with unpredictable challenges. Thus, the biomimetic goal of developmental robotics that is pursued is not to imitate the brain *per se*, namely the performing brain of trained adults, but the process of progressive knowledge acquisition, leading to autonomous decision-making ability by interaction with the physical and social environment.

In particular, we suggest that developmental robotics is intrinsically based on two basic pillars: (1) personal embodiment and (2) personal/social (accumulation of) knowledge. In relation with the first pillar, analyzing the emergence of fetal and neonatal movements Asada et al. (2009) observed that predefined (“innate”) circuits exist in the nervous system: in particular, the stretch reflex in the spinal circuit and the oscillator neurons in the medulla that lead to central pattern generators involved in locomotion; in contrast, it appears that there is no predefined circuit specifying coordination of full-body actions. Thus, it is suggested that the observed synergies of adults emerge from an embodiment process, namely the interaction between the brain and the environment through the body, in the early development stage, and the body model, later on as a result of neural maturation and physical growth. Such internal representation of the body, for describing and predicting the evolution of actions, requires an abstraction from specific sensory modalities (Squeri et al., 2012) and this is the result of a multisensory, multimodal integration-fusion process that is known to occur in late childhood (Chen et al., 2004; Gori et al., 2008, 2012a,2012b; Gori, 2015).

The general principles of developmental robotics are strongly interleaved with the central tenet of embodied cognition, namely that cognitive and behavioral processes emerge from the reciprocal, dynamic and evolving coupling between brain, body and environment throughout the entire life of an individual. This means, in particular, that cognition and intelligence cannot be captured by a complex computer software, such as a deep neural network, because the three-ways interaction mentioned above is intrinsically hybrid, with digital, analogic, and physical components.

As regards the personal/social nature of robot learning/training, we may observe that developmental robots are typically supposed to share the environment with humans, in the framework of a common goal or cooperative task. Therefore, it is beneficial for the robot to infer humans’ goals, imitate their behaviors and/or follow their suggestions in order to accelerate the autonomous acquisition of know-how, marked by the memorization of critical episodic occurrences. Curiosity, exploration and motivation are the essential ingredients to be combined in a synergic way, by linking intrinsic motivation to the attention for the human partner behavior; the role of social environment in this process will be expanded in section 4 “Social support of developmental artificial cognition.”

The crucial role of embodiment, including its ecological aspects, for developmental robotics is apparently challenged by “Vehicles” (Braitenberg, 1986) and the notion of “intelligence without representation” (Brooks, 1991): it is suggested that purposive behavior does not necessarily have to rely on accurate models of the environment, but rather might be the result of the close interaction of a simple control architecture with a complex world. In other words, according to such view there is no need to build enduring, full-scale internal models of the world, because the environment can be *probed* and *re-probed* as needed, thus closing the loop in real-time. More recently, Pfeifer and Scheier (1994) argued that a better global understanding of the perception–action cycle might be required. The authors propose an alternative view that breaking up perception, computation and action into different subsystems might be too strong a commitment. In other words, the *minimal unit of processing* should be *a complete perception–action cycle* that includes the environment in the loop (Vernon et al., 2015b).

We may also add that such computational kernel is likely to evolve during development, with multi-sensory fusion and progressive abstraction from the specific sensory modalities, maintaining and updating an internal eco-body model based on a unifying simulation/emulation theory of action with prospective capabilities (Decety and Ingvar, 1990; Jeannerod, 2001; Grush, 2004; O’Shea and Moran, 2017; Ptak et al., 2017). Clearly, prospection cannot be obtained through the representation-less models of Brooks and Braitenberg that only operate in the present: prospection operates in the future to control the present in a purposive way. It is also worth considering that, despite his emphasis that in general quite complex “intelligent” behavior can be achieved without representation, Brooks accepts a role for representations as building blocks, emerging in a bottom-up fashion (Brooks, 2002). This is a view that matches at the same time the fundamental role of embodied cognition and its ontogenetic evolution as a bottom-up process: internal representations could be introduced at different stages in service for action (Clark, 1997; Steels, 2003). As examples of approaches following such a bottom-up approach we may also quote the DAC architecture (Verschure and Althaus, 2003) or the walking agent model (Schilling and Cruse, 2017; Schilling et al., 2021).

Sensory-Motor-Cognitive Development in humans is an incremental process, involving maturation, integration and adaptation, following the blueprints of a dynamic process moving through more and more skilled cognitive states (Piaget, 1953) and mediated by social interaction (Vygotsky, 1962). However, this process is somehow ragged and the genetically pre-programmed roadmap is steered and implemented by epigenetic phenomena affected by environment. This is related to the long-term discussion of the role of nature and nurture in the development of a personal agent. In other words, development is largely decentralized, event and environment driven, exhibiting the typical features of a self-organizing system. Moreover, there is ample evidence that such spontaneous self-organizing process is aided and amplified through social interactions with adults and peers (Vygotsky, 1962). This includes various types of social support, as scaffolding, guidance, coaching and apprenticeship; moreover, mimetic processes such as mimicry, imitation and emulation are likely to play a central role in cognitive development. More specifically, the development of skilled actions in children can be investigated with three different but complementary approaches that focus, respectively, (1) on the intrinsic maturation of the nervous system (Gesell, 1946), (2) on the information processing aspects, associated with the interaction of the developing nervous system with newly emerging cognitive processes, driven by the interaction with the environment (Connolly, 1970), and (3) on the dynamics of such interaction, i.e., a dynamic systems approach. In summary, it is suggested that the acquisition of new motor skills is guided by the drive to explore (curiosity) and supported equally by the developing nervous system and its interactions with sensory-motor processes and the environment (Bernstein, 1967; Gibson, 1979; Thelen and Smith, 1998; Oudeyer et al., 2016; Schillaci et al., 2016). Moreover, there is mounting evidence (Alderson-Day and Fernyhough, 2015; Fernyhough and Borghi, 2023) that such process of cognitive development is accompanied by the evolution of inner or covert speech that associates a symbolic component to the maturation of subsymbolic sensorimotor capabilities. Such linguistic component has also been linked to the role of inner speech in working memory (Miyake and Shah, 1999), namely the retention of information “online,” critical for a complex task.

If developmental robotics is to be taken seriously in the long run, it should be considered that human cognitive development (approached by means of the theoretical frameworks proposed either by Piaget or Vygotsky) is inexorably interleaved with educational issues and this should be the basis for *training* and *educating* autonomous robotic agents during development. The main motivation and attractive promise for pursuing this line of research is that it may allow to overcome the explainability issue, namely the generalized level of distrust affecting AGI and to give rise to mutual understanding. In other words, we may assume that humans may be readier to trust cognitive robotic agents if they are *well educated* (i.e., robotic agents and humans share a value system), according to clear and public training plans, including such things as a certified CV and reference list. Moreover, by relying on social education and training it will be possible to differentiate the type of skills achieved by the robotic agents in order to obtain some kind of social balance between human and robotic populations as well as to interact differently with human with different abilities (e.g., young or elderly persons).

On the other hand, in spite of the intense and wide research activities on developmental robotics in the last two decades (Min et al., 2016; Huang et al., 2017; Wieser and Cheng, 2018; Naya-Varela et al., 2021; Rayyes et al., 2022), we are still far away from a level of understanding of the global implementation issues that may allow testing and evaluation in a sufficiently general manner. Certainly, there are still limitations regarding the “bodyware” capabilities of robots used to implement a developmental process inspired by humans, including the two most advanced child robots, namely iCub (Sandini et al., 2004) and CB2 (Minato et al., 2008). Their limited sensory and motor abilities is not the most relevant factor so far, to investigate the organization of Sensory-Motor-Cognitive Development in realistic robots, but the fact that most research has been focused on the biomimetic, time frozen implementations of specific aspects of human cognitive development, missing to address the large picture as well as its temporal continuity: in some sense, it is like implementing a specific ability of a three-year-old without figuring out where the system is coming from and how it may progress beyond. This strategy is in contrast with the theory of development, expressed by Vygotsky (1978), describing the “zone of proximal development” as the distance between the actual developmental level and the level of potential development. The actual developmental level characterizes mental development retrospectively, while the Zone of Proximal Development characterizes mental development prospectively. For example, we may quote a representative list of studies in the two preceding decades that cover in isolation some of the specific issues of developmental robotics: Self-exploration and early imitation (Kuniyoshi et al., 2003), Modeling joint attention (Nagai, 2007), Scaffolding Robot Action Learning (Nagai and Rohlfing, 2009), Affordance-based perception (Min et al., 2016), Bootstrapping the semantics of tools (Schoeler and Wörgötter, 2016), Perception of Localized Features (Giagkos et al., 2017), Bootstrapping of Sensory-Motor Skills (Wieser and Cheng, 2018), Developing Reaching Ability like human infants (Luo et al., 2018), Sensorimotor Communication (Donnarumma et al., 2012, 2018; Pezzulo et al., 2019), Integration of Sensing, Cognition, Learning, and Control (Li et al., 2019), Evaluation of Internal Models (Smith and Herrmann, 2019), Emergence of symbolic representations (Ugur et al., 2015; Taniguchi et al., 2019), Grounded affordances (Saponaro et al., 2020), Bodily Expression of Emotion (Tuyen et al., 2021), Morphological development (Naya-Varela et al., 2021), Skill Learning Strategy with Dynamic Motion Primitives (Li et al., 2021), Interest-driven exploration (Rayyes et al., 2022, 2023).

This “stroboscopic” view of development, even if advancing the field, completely misses the continuous dynamic process behind sensorimotor and cognitive development. To advance the field of developmental robotics we should focus on a wider view of cognition, making explicit the relationship between different cognitive skills and their shared functions, although we should not disregard bodyware technologies because, obviously, better bodies can be advantageous.

In spite of the intrinsic fragmentation of the growth/integration process that needs to be overcome to give developmental robotics its full potential, the alternative roadmap that aims at the design of fully developed “adult” cognitive architectures for autonomous robots is only apparently more direct and simpler: in contrast, we believe that it misses the crucial fact that it is precisely this being always “under construction” that characterizes human intelligence and its open cognitive development.

A recent review of forty years in Cognitive Architecture Research for autonomous robotics (Kotseruba and Tsotsos, 2020) clearly shows that we are still far away from a solid platform. The problem is that it does not make sense to aim at a General-Adult Cognitive Architecture that (as the dream of AGI) can be easily adapted to any application area; at the same time, it is not very attractive to aim at a population of cognitive architectures, specifically designed for each application, without a well-defined common computational core. In contrast, the developmental roadmap, which is apparently more arduous, would take advantage of a close interaction with humans and the well-developed human tools for building, communicating and transmitting knowledge and culture (Vernon, 2014).

One of the crucial problems that will challenge in the future the research in developmental robotics might be the identification of a minimal set of sensory-motor-cognitive kernels capable to bootstrap the growth, through self-organization, interaction, and training, of sensory-motor-cognitive abilities optimized for different application areas. With respect to this minimal set it has become clear that the apparent simplification offered by the analogy to human development, characterized by an incremental improvement of cognitive skills, on one side reduces the complexity of the learning processes by suggesting the sequence of the functions to be learned but on the other does not make explicit the complexity of the underlying computational/functional architecture of the system which is fully formed during the gestational period and expressed through highly structured brain connectivity. The baseline architecture represents the blueprint of evolution providing the scaffolding for innate behaviors as well as for continuous learning (Zador, 2019). It is the opinion of the authors that without a baseline cognitive architecture with the potential to express all cognitive functions the advantage offered by a developmental approach to the design of ACo system will continue to produce temporally and functionally fragmented robots. It is worth mentioning that this baseline cognitive architecture is not the result of learning but is encoded in the genome and marginally modified epigenetically and, therefore, in ACo systems may require different computational tools than those used during the successive developmental and lifelong learning phases (among other differences its structure is only marginally affected by sensory and social interaction).

From the computational point of view, we may suggest that the cognitive architecture of cognitive agents will not be a closed piece of software running on a powerful but traditional von Neumann computer: in contrast, it can be conceived as a hybrid dynamical system that changes over time as an effect of learning and on-line interaction with humans or other cognitive agents. This growth process may take advantage of a number of possible tools, for example using neuromorphic computing technologies that may match better than traditional von Neumann design the need of self-organized growth (Tang and Huang, 2018).

**FIGURE 1:**
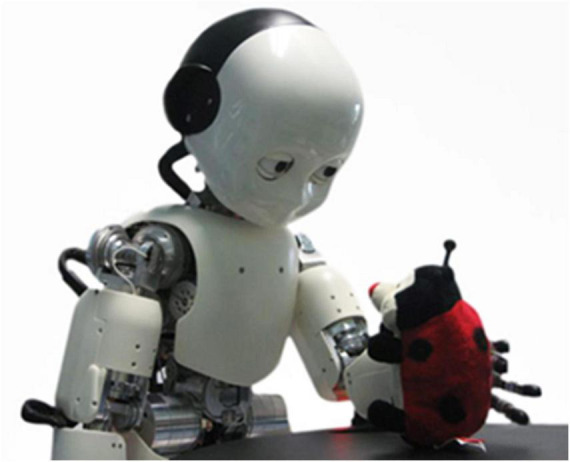
iCub: the first baby robot.

Moreover, we expect that the converging integration of technologies and methodologies will be facilitated by the development of the next generation of baby robots, following the first baby robot iCub (Figure 1) with better sensory-motor-cognitive capabilities.

## 3 Body model and prospection capabilities for artificial cognition

Evolutionary pressure is behind the increase and expansion of motor redundancy in the human species, with emphasis on two functional areas: manipulation (through the availability of thumb opposition) and vocalization (through the development of a sophisticated vocal tract). Such bodily evolution has been accompanied by the emergence of specialized brain regions required to tame the abundance of DoFs, with a dual computational function: control of *overt* (real) actions and generation of *covert* (imagined) actions for allowing adult subjects to reason in a proactive way, in agreement with the fundamental cognitive capability of *prospection* (Gilbert and Wilson, 2007; Seligman et al., 2013; Vernon et al., 2015a,b). Prospection includes planning, prediction, imagination of hypothetical scenarios, and evaluation/assessment of possible future events. Prospective abilities – how much and how well a person is able to bring mental representations and evaluations of possible futures to bear on the selection of action – is a fundamental cognitive faculty akin to other basic faculties as language and reasoning.

The key achievement for cognitive mastering the physical interaction with the environment is prospection, which can also be described as “Mental Time Travel” (Suddendorf and Corballis, 2007) in order to emphasize the extended role of memory in purposive actions: episodic memory (retrieving from the past events/situations, specifically relevant for the current state) as well as procedural memory (namely, the identification of the appropriate selection of tools and sub-actions as well as the prediction of the perceptual feedback); such memorized know-how can be combined for imagining future scenarios and evaluating alternative courses of action. Mental time travel allows cognitive agents to act skillfully by integrating and fusing in the performed action the past (through smart retrieval of goal-driven experiences), the present (through the activation of previously trained synergies), and the future (through the anticipated internal and external consequences of the action). Moreover, the propensity for prospection and mental time travel can be greatly enhanced by another intrinsically social mental ability, i.e., flexible communication and language, enhancing the potential predisposition of humans to cooperate by pooling information and resources (Tomasello, 2009; Slocombe and Seed, 2019).

In relation with the unifying simulation/emulation theory of action (Decety and Ingvar, 1990; Jeannerod, 2001; Grush, 2004; O’Shea and Moran, 2017; Ptak et al., 2017) we suggest that the basic computational module required for a cognitive agent to achieve prospection is an internal representation of the whole body or body schema. In neuroscience “body schema” is a label (with other similar labels, as body image etc.) that covers a scattered range of phenomena rather than a specific well-modeled neural mechanism (Head and Holmes, 1911; Penfield and Boldrey, 1937; Gallagher, 1985; Graziano and Botvinick, 1999; Paillard, 1999; Holmes and Spence, 2004; Preester and Knockaert, 2005; Hoffmann et al., 2010; Morasso et al., 2015; Di Vita et al., 2016). However, there is an agreement on some computational features that are relevant for the development of cognitive robotic agents: the body schema (1) is spatially encoded (with multiple reference frames), (2) is intermodal/supramodal (including the dynamic integration of sensory and motor information (Azañón et al., 2016), (3) is distributed and modular (in multiple cortical maps dynamically interconnected into networks such as the “default mode network”) (Horn et al., 2013), (4) is characterized by a short-term plasticity and reorganization on the time scale of seconds, as shown by the quick integration of tools into the body schema for skilled performers (Maravita and Iriki, 2004).

**FIGURE 2:**
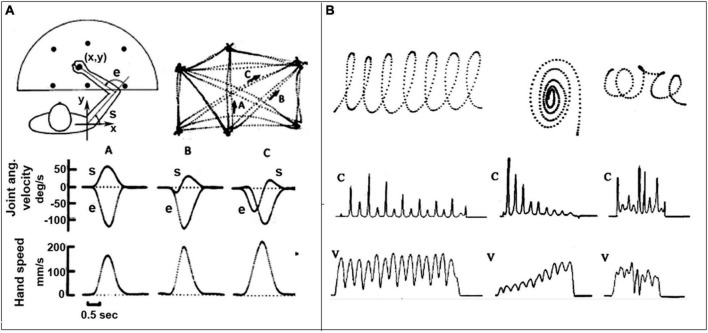
Spatio-temporal or figural-kinematic invariants in trajectory formation. **(A)** Planar reaching movements between six target points; note the invariant straight point-to-point trajectories and the invariant bell-shaped speed profiles. **(B)** Three examples of continuous hand gestures displayed as digitized trajectories, including the profiles of the velocity (V) and curvature (C); note the anti-correlation of the two profiles. From Morasso, 2022.

Moreover, there is ground to believe that the body schema is directly responsible for the kinematic-figural invariants that characterize the spatio-temporal features of biological motion (Johansson, 1973). Such invariants are mainly related to the spatio-temporal features of the end-effectors rather than joint coordination, suggesting that the brain representation of action is more skill-oriented than purely movement/muscle-oriented. For example, Figure 2 shows that in reaching movements the trajectory of the end-effector is straight with a symmetrical bell-shape of the corresponding speed profile, independent of the starting position, direction and of the specific end-effector; in whole body gestures, as handwriting or drawing, the curvature and speed profiles are anti-correlated (Morasso, 2022) and characterize what is known as “biological motion” (see below). Since these invariants are independent of the number of DoFs recruited in a given action, we may suggest that the computational machinery producing them is actually solving, at the same time and in an intrinsic manner, what Bernstein (1967) called the Degrees-of-Freedom-Problem.

It is also worth considering that the study of motor imagery provided evidence that kinematic invariants are also present in covert movements (Decety and Jeannerod, 1995; Karklinsky and Flash, 2015). Such evidence, together with the finding that the same invariants characterize the actions of congenitally blind persons (Sergio and Scott, 1998), suggests to focus the attention of the computational analysis of kinematic invariants from their phenomenological description [e.g., the minimization of jerk (Flash and Hogan, 1985) or the 2/3 power law (Lacquaniti et al., 1983)] to the organization of internal models incorporating the intrinsic dynamics of the bodyware.

The Mental Simulation Theory (Jeannerod, 2001) provides a powerful approach to the solution of this problem: it posits that the brain generates a mental flow of simulation-states in such a way as to simulate the activity produced during the same, executed action and this process “would put the action representation in a true motor format so that it would be regarded by the motor system as a real action.” A computational implementation of the mental simulation theory that can be easily integrated in the cognitive architecture of autonomous robots is the PMP model of trajectory formation (Passive Motion Paradigm: Mussa Ivaldi et al., 1988, 1989; Mohan and Morasso, 2011; Mohan et al., 2019): its basic rationale is the same as the models of action representation and motor control based on a force-field approach, namely the idea that the motor coordination of multiple, redundant DoFs of the body is the consequence of (real or virtual) force fields applied to an internal representation of the body (the body model). The body model is viewed as a network of spring-like elements that individually store elastic potential energy, contributing to a global potential energy that recapitulates, in a smooth, analogic manner, the complex set of bodily interactions: the network is then characterized by an attractor neurodynamics that aims at equilibrium states of minimum potential energy, in a similar way to the dynamics of Hopfield networks (Hopfield, 1982). The PMP model applies the concept of *passive* motion to *active* synergy formation by updating the control input of each element of the body model so as to cancel the “stress” induced by a simulated external perturbation, e.g., the attractive force field to a designated target.

There is also an interesting relationship between the Passive Motion Paradigm and Active Inference (Friston et al., 2011): the anti-symmetry between *active inference* and *passive motion* speaks to the complementary but convergent view of how we use our forward models to generate predictions of sensed movements. This view is an example of Dennett’s “strange inversion” (Dennett, 2009), in which motor commands no longer cause desired movements – but desired movements cause motor commands (in the form of the predicted consequences of movement). Thus, the PMP model can tame the abundance of degrees of freedom of the human body by using a small number of primitives (force fields, associated with specific end-effectors as a function of a given whole-body gesture): the diffusion of such fields throughout the internal body model distributes the activity to all the DoFs, with an attractor neurodynamics, driven by the instantiated force fields, that indirectly produces at the same time the kinematic invariants mentioned above for both the overt and covert actions.

At the end of the ontogenic development of adult subjects, we may assume that the redundant degrees of freedom of the human body are coordinated by the brain in such a way to exhibit the observed kinematic invariants by animating the body schema according to something similar to the PMP computational model. From what is known of the sensory-motor-cognitive development of humans we may exclude that the organization of the body model is somehow innate and genetically preprogrammed^3^. In contrast, we may expect that it is built and refined during development by exploiting plasticity, consolidating a physical self-awareness and conquering two crucial developmental targets; (1) multi-modal sensory fusion and calibration, for achieving perceptual abstraction (Gori et al., 2008, 2012a) (2) generalized kinematic invariance, for general end-effectors and/or skilled tool-use.

**FIGURE 3:**
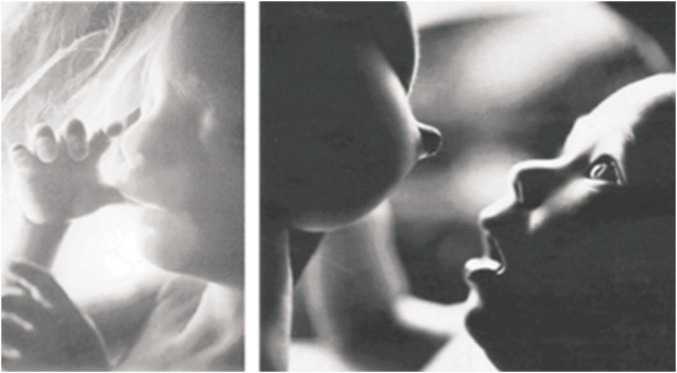
Pre-natal and post-natal visuo-haptic experience.

As regards self-awareness/self-body-consciousness, it has been suggested (Tononi, 2004) that it is the result of a general process of information integration carried out by the brain through the neural connectome (Tononi, 2005) and, from a developmental point of view, is an emergent property of the interaction between brain, body and environment, with the crucial role of visuo-haptic information (Morasso, 2007). Infants spontaneously touch their own body and reach to tactile targets on the skin already in the final part of pregnancy and with even more intensity in the post-natal period (Figure 3), when touching and being touched occurs systematically, with the reinforcement effect of mother care (Corbetta and Snapp-Childs, 2009; Di Mercurio et al., 2018): infants are active explorers of their own body as well as of their peripersonal space (Cardinali et al., 2009) from the first days of life and such self-generation of multi-sensory information is likely to shape and adapt Tononi’s connectome. This primordial and self-generated sense of self-unity is suggested to be the crucial initial base from which learning and development may evolve (Rochat, 2019). However, the self-exploration mentioned above in early childhood, although crucial for building a solid awareness for the emergence of a “body space,” with the surrounding “peri-personal space,” as well as a strong sense of unity is initially rather qualitative and sensory-modality dependent. As shown by Gori et al. (2008), children before 8 years of age fail to integrate visual and haptic spatial information. More generally, Gori (2015) proposed a cross-sensory calibration theory, according to which before 8−10 years of age the more accurate sensory modality “teaches/calibrates” the more variable sensory source and only afterwards accurate and reliable multisensory information is made available by the CNS. In contrast, adults are able to optimally fuse multisensory information in an abstract form (Ernst and Banks, 2002). An effective internal representation of the body, for describing and predicting the evolution of actions, requires an abstraction from specific sensory modalities as well the corresponding motor information flow (Squeri et al., 2012).

As regards the emergence of kinematic invariants, as indicators of the consolidation of the body schema, it is worth considering the maturation of the kinematic patterns of reaching movements. First of all, it has been observed that although infants reliably grasp for objects within their workspace 3−4 months after the onset of reaching, stereotypic kinematic motor patterns begin to emerge between the 2nd and 3rd year of life (Konczak et al., 1995; Konczak and Dichgans, 1997; Gonçalves et al., 2013; Zhou and Smith, 2022): for example, only at that stage, the majority of trials exhibit a single peaked velocity profile of the hand. The observation of reaching movements beyond the 3rd year of life (Schneiberg et al., 2002) reveals that development is characterized by a gradual decrease of the variability of Interjoint coordination and end-point trajectories and an increase of their smoothness: the highly stereotyped and stable patterns that characterize adults are reached only after 10−12 years of age and probably further consolidated in early adolescence (Mitchell, 1998).

In general, the concept of body schema implies an internal awareness of the body (Berlucchi and Aglioti, 1997) and the relationship of body parts to each other, encompassing cognitive aspects. In the framework of the Piaget theory of cognitive development that identifies four stages of maturation, the preliminary consolidation of the body schema as a working computational mechanism may be located at the end of the third stage (*concrete operational thinking*, at the age of 11−12 years) that marks the beginning of abstract or operational thought: this means that the child can imagine things/situations internally in the mind, rather than physically try things out in the real world. However, such *operational thought* is only effective if the child is asked to reason about materials that are physically present. Only afterwards, at the later stage of *formal operational thinking* while entering adolescence, children may gain the ability to think in a more abstract and systematic manner, reasoning about what might be as well as what is, as an effect of their actions and the related reactions of the environment. As a matter of fact, such fourth stage of Piaget’s theory is open-ended and strongly dependent on the specific interaction with the physical/social environment as a life-long learning/training process (Flesch et al., 2023).

In summary, we suggest that the Body Schema (animated through the mental simulation theory) and the Prospection capabilities, which are essential for the Artificial Cognition of the next generation of cognitive robots, may be achieved more naturally in a developmental, self-organized framework. The achievement of this goal will probably require the adoption of new computing technologies, beyond the traditional von Neumann approach, allowing the implementation of a kind of *large connectome* as the basic skeleton for the cognitive development of the robot. The connectome may support the emergence of a number of self-organizing maps, derived from the pioneering research of Kohonen (Kohonen, 1982; Martinetz and Schulten, 1994): this self-adaptive neural substrate, evolving under the action of Hebbian learning mechanisms, should be driven by a built-in, spontaneous tendency to explore the body space and the peripersonal space: a generalized, spontaneous babbling strategy (Kuperstein, 1991; Goldstein and Schwade, 2008). Babbling is a stage in child development (4−12 months of age) and a precursor to language acquisition: infants experiment with *phonation* by uttering sounds that not yet produce recognizable words but are essential to self-generation of training data for the phonatory system. A similar babbling strategy appears to be in operation in the same period, for experimenting *manipulation* and acquiring self-awareness of the body that initiates the construction of the body schema. Thus, we suggest that such generalized babbling mechanism, ranging from phonation to manipulation, is a crucial bootstrap mechanism for cognitive developmental robotics. At the same time, the maturation of prospection capabilities via the evolution of mechanisms for the mental simulation of covert actions can be integrated with the evolution of covert and overt speech (Alderson-Day and Fernyhough, 2015) that associates a symbolic component to the maturation of subsymbolic sensorimotor capabilities. Moreover, reinforcement learning (Neftci and Averbeck, 2019) can support the strategic organization of purposive actions for cooperative human-robot interaction.

## 4 Social support of developmental artificial cognition

For cognitive robots conceived in the framework of developmental robotics, cognitive capabilities are not preprogrammed but can be achieved and organized through interaction with the physical and social environment.

While the former sections have addressed the development of cognition mostly focusing on an individual agent, the current one underlines the importance of the social dimension and advocates the need of considering it since the beginning of the modeling process of artificial cognition. This claim draws parallels with human cognition, where the innate predisposition of humans for social interaction from early stages influences cognitive, emotional, and motor development. Then, a pathway to model core cognitive abilities in artificial agents based on a social perspective is proposed. It starts from the incorporation of social motives into artificial cognitive systems, moves on emphasizing the impact of bidirectional non-verbal linguistic interactions for effective cognitive development in robots and concludes by highlighting the role of social interaction in consolidating cooperation, teamwork, and the transmission of ethical norms and cultural values in cognitive agents.

Human beings show a marked predisposition to work together toward joint goals and in cooperation, to teach (and be taught by) others and to consider their perspectives and intentions (Tomasello, 2009, 2018; Slocombe and Seed, 2019). It has even been proposed that human beings have evolved special social-cognitive skills beyond those shared by primates in general for living and exchanging knowledge in cultural groups, by communicating, learning and “reading the mind” of others in complex ways (Herrmann et al., 2007). In particular, the generalized capacity for teaching, which involves the use of shared attention and other forms of verbal and non-verbal information exchanges, is one of the features at the basis of the extreme capacity for cultural learning shown by humans (Laland and Seed, 2021).

The predisposition to social interaction is apparent since the initial moments of human life. Already before birth, the direct interaction with the mother and, when present, with the twins (Castiello et al., 2010) and with the social inputs coming from outside the womb (e.g., as sounds) play an important role in shaping the future interaction of the baby with the world and others (Ciaunica et al., 2021).

Since birth, human neonates are attracted by other people and are endowed with skills that facilitate the establishment of an interaction, ranging from the preference for biological motion (Simion et al., 2008) and for faces looking directly to them (Farroni et al., 2002), with preference for human voices to other sounds (Alegria and Noirot, 1978) and especially infant directed speech (Cooper and Aslin, 1990). As soon as about half an hour after birth newborns can imitate facial expressions of happiness and surprise (Meltzoff and Moore, 1977, 1983; Field et al., 1983) and within the first following days, newborns look significantly longer at a happy facial expression than a fearful one, suggesting a sensitivity to the facial characteristics that maximize their chances of interacting with others (Farroni et al., 2007).

It appears that particular high relevance is given since the earliest moments of development to elements that ensure the affiliation with conspecifics. This is not unexpected in a species where newborns are born with undeveloped skills and strongly depend on the support of their caregivers to survive and grow in the first portion of their life (i.e., an *altricial* species, in contrast to *precocial* ones (Vernon, 2014).

Such social inclination at birth is not the result of supervised or unsupervised learning algorithms but an inherited predisposition to exploit social interaction for the development of cognitive abilities through the production and understanding of social signals, such as gestures, gaze direction and emotional displays. It is worth mentioning here that some of these innate “social signs” are in fact exapted functions (Gould and Vrba, 1982) in the sense that the main motivation they appeared during evolution was not for their “social use.” For example, facial expression (Murray et al., 2017) are supported by part of the system for regulating ingestion in relation to breathing and the need to control the direction of gaze is mainly motivated by the computational advantages of human space variant retina (Sandini and Tagliasco, 1980). Starting from these innate skills the social competence of the newborn develops very rapidly. Around 3 months of age infants engage mutual gaze with adults, i.e., both agents attend to each other’s eyes simultaneously (Kaplan and Hafner, 2006). At around 6 months of age infants can perceive the direction of attention of others, at least in term of discriminating whether the caregiver’s gaze is directed to the left or to the right (Butterworth, 1991; Butterworth and Jarrett, 1991). In the sensitive period for social coordination, from 3 to 9 months of age, parents and infants establish social interaction by coordinating gaze, affect, vocalizations and touches. These reciprocal exchanges shape the child’s development, impacting not only the social domain, but also the emotional, cognitive and brain development (Feldman, 2007, 2012).

By the end of their first year of life infants start to understand pointing as an object-directed action (Woodward and Guajardo, 2002) and to address it, their gazing and vocalizations more often to people than to inanimate objects, showing that they are aware of the person’s attentional state (Legerstee and Barillas, 2003). Still during the first year of life infants demonstrate action understanding skills strongly correlated with the infants’ prior motor experience (Sommerville and Woodward, 2005; Sommerville et al., 2005). For instance, the capability to execute and anticipate reaching (Kanakogi and Itakura, 2011) or eating actions (Kochukhova and Gredebäck, 2010) appears at 6 months-of-age, while at least 9−12 months of life are required in order to skillfully perform and anticipate the goal of a transport action (Falck-Ytter et al., 2006).

Instrumental helping emerges already at 18 months of age, when toddlers altruistically try and help adults when they don’t manage to reach a certain goal (Warneken and Tomasello, 2009) and then increases its complexity and selectivity with age (Slocombe and Seed, 2019).

The presence of innate skills and the rapid development of early abilities suggest a preparedness for social interaction and a primary importance of social relationships in the development of cognition. Such importance is confirmed also by studying the behavior of caregivers, as it has been proven that maternal postpartum behavior shapes children’s symbolic competence and cognitive skills (Feldman et al., 2004; Feldman and Eidelman, 2009). Immediately after birth human mothers express specific newborn directed behaviors, such as gazing at the infant’s face and body, expressing positive affects, modulating their prosody by using high-pitched vocalizations (“motherese”), and affectionate touch. Importantly – and proper for the human species only – mothers show reciprocity, i.e., mothers adapt their behavioral intensity to the neonate’s moments of alertness (Feldman and Eidelman, 2003, 2007).

These findings suggest that, when attempting to model cognition, it is mandatory not only to address the individual agent and its relation with the environment, but also to consider the impact of being immersed in the social world and somehow predisposed to social interaction since the earliest moments of development. In this perspective, the necessity to thrive in a social context becomes a foundational element of how the agent’s cognition develops and of which are the basic abilities with which it needs to be endowed first.

A similar shift has recently informed the field of neuroscience with the call for a change in paradigms toward the so-called “second-person neuroscience” (Schilbach et al., 2013), in which neural processes are examined within the context of a real-time reciprocal social interaction. Neuroimaging and psychophysiological studies have provided evidence that social cognition is fundamentally different when someone is involved in an interaction and emotionally engaged as compared to being just an observer (Redcay and Schilbach, 2019).

Also studying artificial cognition could benefit from shifting from modeling of the individual agent and of its (passive) understanding of the social environment to a perspective where active social interaction is foundational and transformative, impacting the way the agent faces the complex problem of understanding and anticipating others and its cognition overall. Rather, approximating the development of cognition as sequential process where the agent first develops in isolation certain skills and then merely exploits them in its interaction with others might lead to overseeing some crucial components of what makes human-like cognition so advanced and capable of generalization.

Some authors have even proposed that social interaction might be the brain’s default mode (Hari et al., 2015). Indeed, although social interaction represents one of the most complex functions that human cognition enables and one of the most difficult to implement on cognitive artificial agents, still it appears to us incredibly easy. A paradigmatic example is that of dialogue: turn taking during human-human conversation is naturally achieved effortlessly and with an extreme temporal accuracy, yet unmatched in human-agent verbal exchanges. It has been suggested (Garrod and Pickering, 2004) that humans are “designed” for dialogs, endowed with unconscious interactive processing, seamlessly aligning the linguistic representations of the interlocutors. Furthermore, the evidence that children learn best during interaction than by observing others (Moll and Meltzoff, 2011) has been proposed as another evidence in favor of the primacy of interaction (Hari et al., 2015).

This is not to deny the principle that individual first-hand competence (e.g., action execution) plays a crucial role in shaping the understanding of the social world, which has received very strong support also from neuroscience. As an example, it has been proven that children have first to learn how to perform an action themselves before being able to automatically anticipate the outcome of the same action, when it is executed by another agent (Falck-Ytter et al., 2006). This process maintains its validity also during adulthood (Flanagan and Johansson, 2003). It’s only through extensive first-hand motor experience that athletes become extremely proficient at predicting the outcome of others’ actions in the same sport, e.g., guessing whether a basketball will enter the loop or whether a certain kick will lead to a goal. Sport journalist, though accumulating a similar amount of observational exposure, do not reach comparable anticipatory capabilities (Aglioti et al., 2008).

However, the centrality of the reciprocity in infant-caregiver behaviors in the development of children cognition (Feldman and Eidelman, 2009) and the evidence that a great component of human learning is inherently social and (almost uniquely in the human species) relies on teaching (Csibra and Gergely, 2011; Laland and Seed, 2021), indicate that the traditional approach of “developing the individual” and then face it with the social environment could lead only to a partial comprehension of human-like cognition. Citing Hari et al. (2015) *“If the goal is to achieve human-like behavior, it is not enough to build on the bottom-up stimulus-driven effects, but the centrality, eventually primacy, of social interaction should be incorporated to the models*.”

Following this approach, it would be worth analyzing the core cognitive capabilities currently identified for Cognition (Perception, Attention, Action Selection, Memory, Learning, Reasoning, Metacognition and more recently Prospection: Vernon et al., 2007; Vernon et al., 2016; Kotseruba and Tsotsos, 2020) through a “social lens”: how they developed based on the necessity of being a social agent in a social world?

As a starting point, we may include social motives in the development of an artificial cognitive system. A motive defines the goal of the system and determines its decision making. The social motive guides the agent toward the research of comfort, security and satisfaction from the interaction with others, guiding to the exchanges of information and to the need of maintaining the interaction over long periods of time (Vernon, 2014). This approach is being explored in some cognitive architecture. For instance, in the Clarion modularly structured cognitive architecture (Sun, 2006) the motivational subsystem derives goals both from physiological needs (such as need for food, need for water, need to avoid danger, and need to avoid boredom) and so-called high-level drives, such as desire for social approval, desire for following social norms, desire for reciprocation, and desire for imitation of other individuals. More recent attempts in robotics have looked into social motivation where the need for social comfort drives the action selection and adaptivity (Hiolle et al., 2014; Tanevska et al., 2020; Mongile et al., 2022). Including social elements, e.g., social norms, in the development of basic learning skills of an agent, is another step in this direction such as considering social constructs such as rivalry when learning to win a competitive game (Barros et al., 2022) or ensuring legibility of the agent’s reinforcement learning process for the human teacher (Matarese et al., 2021).

From the point of view of developmental cognition, we should then emphasize the critical role of pedagogy that is supposed to integrate subtle scaffolding, motivation, teaching, training and information transmission for children of primary school age (Csibra, 2010; Csibra and Gergely, 2011). In particular, this also includes a combination of verbal and gestural/bodily linguistic interactions between the teacher and the pupil, aimed at grabbing and directing the attention of the pupils: for example, effective gestural language refers to pointing, with the fingers and/or the eye, and adopting facial expressions for expressing surprise, curiosity, interest, fear etc.

**FIGURE 4:**
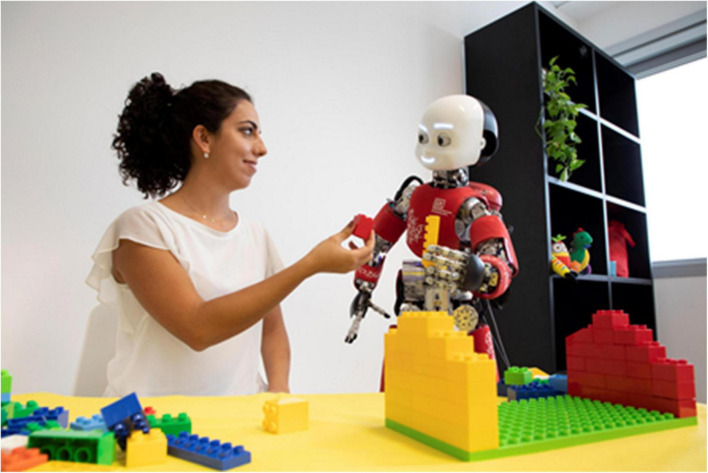
Mutual understanding, supported by the exchange of verbal and non-verbal signals, and a reciprocal interaction with the caregiver, are at the basis of children’s cognitive development. They might be as necessary for the development of Artificial Cognitive systems.

Developing systems that can understand and properly express such wide range of implicit cues (Figure 4) becomes then crucial in facilitating the overall development of the skills of the cognitive system, not only of its social competences (Sandini and Sciutti, 2018; Sciutti et al., 2018). Considering that in humans simpler forms of sensorimotor communication might have scaffolded more complex cognitive abilities, such as linguistic communication, endowing robots with sensorimotor communication abilities might aid in developing more advanced interaction capabilities Hence, the need of developing systems that are sensitive to the subtle variations of human movements of face, body and gaze (Palinko et al., 2016; Vignolo et al., 2017; Cazzato et al., 2020; Barros and Sciutti, 2022; Barros et al., 2022) and that can embed in their own behavior the same subtle cues that make human action seamlessly understandable to the human partner (Di Cesare et al., 2020; Lastrico et al., 2023).

Moreover, such non-verbal linguistic interactions are strongly bidirectional: from the child to the adult and vice versa. Thus, if we aim at promoting the maturation of the cognitive abilities of developing robots we should refer more to pedagogists rather than to ICT experts.

At later stages of development, the role of social interaction should focus on consolidating the tendency of the cognitive agent to cooperate and integrate in a teamwork (Tomasello, 2009), including the ability to represent the mental states (both of oneself or partners) and communicate/talk with the partners about knowledge, beliefs, desires, intentions, and so on. More generally, the reliability of ACo robots in the social context is determined by a shared value system that, to a great extent, can emerge from structured social interaction, capable to transmit ethical norms and cultural values (Boyd and Richerson, 1985; Tomasello, 1999). Obviously, this is a highly sensitive issue that requires a great care and responsibility. Moreover, language is certainly a crucial tool in this process, both the inner speech for consolidating and retrieving sensory-motor-cognitive capabilities and the open speech for allowing cognitive agents to reason about cooperative tasks and openly explore different alternatives. Moreover, here is a possible link between ACo and AI in the sense of using LLMs as well-developed working tools.

## 5 More on ACo vs. AI in relation with cognitive neuroscience

After having explained in detail the main pillars of the ACo framework, it is worth explaining the differences between ACo and other attempts inspired by cognitive neuroscience and somehow linked to AI. In particular, comments are provided for clarifying why we believe that attempts to extend current AI in the sense of cognitive neuroscience miss crucial points. Such attempts are motivated by a preliminary, fully acceptable consideration, that current AI applications (shortly AIs) do not meet social expectations in many cases: for example, when a specific AI-based agent faces situations that their designers did not anticipate the result is failure or, even worse, irrational and unexplainable behaviors that evidentiate lack of understanding and ineffective interaction/cooperation with people or other agents. As regards the attempts to make AIs more compatible with neuroscience we already mentioned the issue of **Explainable AI** (XAI), namely the “explainability” problem of AI technologies, and the proposed solution (Taylor and Taylor, 2021; Iglesias, 2022) to import assessment methods developed in Psychology and Cognitive Neuroscience: although this may be a useful method of analysis, it is of no help from the “generative” point of view, i.e., for the design of autonomous, prospective cognitive agents. Another stream of neuro-inspired AI is **Embodied AIs** (Duan et al., 2022): please, note the plural, with the implicit assumption that Intelligence or Cognition is not the fundamental organizational principle from which all the specific competences are derived and integrated but is rather the result of the mere assembly of specific and substantially independent AIs, like perceiving, skilled gesturing, abstraction discovering, planning, modeling other agents, self and social awareness, interacting, and communicating. Embodied AI research diverges from conventional AI where learning is orchestrated on collecting big data and using them separately for different functions (perception, motor control, planning, etc.) with a third-person (impersonal) view of the acquired knowledge. In contrast, embodied AI is characterized by a first-person perspective that aims at mimicking human behavior during the interaction with the environment. However, this view of Embodied AI captures only a marginal part of Embodied Neuroscience: for example, in the radical formulation of the embodied brain (Kiverstein and Miller, 2015) it is suggested that cognitive neuroscience should look to an ecological dynamical psychology where the functions associated to brain regions are dynamically changed over time and are based on the inseparability of cognitive and emotional processing; emotional states should be best understood in terms of action readiness (both overt and covert actions) in the context of the organism’s ongoing skillful and prospective engagement with the environment. States of action readiness involve the whole living body of the organism, including a dynamic body schema, and are elicited by possibilities for action in the environment (more generally *affordances*) that matter to the organism. Thus, the egocentric characterization of Embodied AI captures only a small part to the fundamental properties of Embodied Cognition that allow a cognitive agent to operate in a successful and autonomous way in a changing and unpredictable environment: moreover, switching from a first-person to a third-person perspective and back is one of the crucial powerful cognitive features of prospection.

Let us consider another possible variation of neuro-inspired AI, namely **Developmental AIs** (Stefik and Price, 2023) aimed at bootstrapping a process that may evolve from simple innate competences to intelligent, human-compatible AIs. The premise of both Embodied AIs and Developmental AIs is that a better approach for AIs to acquire what knowledge is needed for a given task is by observing and probing the environment, interacting with people and with socially developed information including online media. Moreover, bootstrapping developmental AIs is about creating socially aware human compatible AIs that learn from and teach others. It is suggested an approach that follows a bio-inspired trajectory for bootstrapping separately the different AIs: perceiving, understanding and manipulating objects; multi-step actions and abstraction discovery; curiosity and intrinsic motivation; imitation learning; imagination and play; communication and language. The bootstrapping approach tracks a competence trajectory where the competences are developed in small steps in parallel by embodied AIs. Although such items are certainly relevant in developmental robotics, developmental AI ignores/under evaluates a key issue, i.e., the fact that development is a self-organizing process where the different functions or AIs cannot be separated with the classical “divide and conquer” strategy. Both the proposed Embodied AIs and Developmental AIs implicitly assume a separation of Bodyware and Cogniware where Cogniware consists of a collection of software packages running on traditional von Neumann computational architectures. The Artificial Cognition approach supported in this work suggests a full integration of Bodyware and Cogniware, with a deep compatibility between artificial and human cognition that implicitly solves the explainability problem providing a common interaction language. Moreover, ACo is not the mere collection of Embodied AIs and Developmental AIs but is brain-inspired to the extent that Embodied and Developmental features are essential parts of its DNA. Thus, the cognitive architecture embedded and embodied in the Cogniware will provide a coherent baseline to facilitate convergent, cumulative progress in the development of an operational model of cognition and in parallel will facilitate experimental testing of core cognitive abilities and their dynamic and synergistic interplay as the robot interacts with its environment and other cognitive agents.

## 6 Conclusions

There is wide agreement that understanding the brain structure and function is one of the most substantial and challenging frontier scientific questions of the 21st century, as clearly expressed by several world-wide proposals of “brain projects” in the last two decades (Jones and Mendell, 1999; Markram, 2006; Kandel et al., 2013; Okano et al., 2015; Poo et al., 2016; Ramos et al., 2019; Bjaalie et al., 2020; Yuan et al., 2022). In most cases, such projects have a double target: a medical target and a computational target. For example, in (Yuan et al., 2022) it is stated that “the China Brain Project is structured as “one body and two wings”, with the goal of developing treatments for major brain disorders and promoting the development of a new generation of artificial intelligence.” While in the last two decades the research in the “first wing” has produced a number of interesting and useful results, the “second wing” is still far away from any preliminary conceptual framework for a bio-inspired and neuro-driven approach to the design of autonomous, cognitive, robotic agents: on one side, the current wave of AI research and development is totally dis-embodied and disconnected from any brain-like formulation and, on the other, the brain projects mentioned above do not really address the cognitive/computational issues in depth. For example, the EU-funded Human Brain Project has ended without fulfilling its promise to build an artificial simulation of the brain that could support the second “wing” of brain-related projects in a significant way.

As we discussed in this paper, we are convinced that the future generation of autonomous robotic agents supporting humans in everyday activities should be strongly neuro-inspired, not in the sense of exploiting the detailed simulation of the biophysics of the adult human brain but of exploiting the processing structure and functions supporting human ability to act prospectively and interact with others on the basis of mutual understanding. In relation with the general issues of the brain projects mentioned above it is worth considering briefly the relationship between the cognitive abilities of the human species and animals, with particular reference to monkeys and chimpanzees. Although the wide spread prejudice of human superiority, based on the contrast between human rationality and animal instinctuality, research into animal cognition (Gallistel, 1989; Laland and Seed, 2021) has established striking similarities for all the typical cognitive abilities, such as memory, problem solving, tool construction and use, communication, and social interaction with proven superiority in short-term memory in chimpanzees (Matsuzawa et al., 2006). The difference is more in the degree of sophistication and generalization, although there is still some debate on the ability to implement mental time travel by animal species (Clayton et al., 2003; Suddendorf and Corballis, 2007). Maybe, we may consider cognitive robots as representative of an artificial humanoid species to be epigenetically designed, grown up, trained, and educated in such a way to be compatible with humans, human society, and human cultural values.

The detailed simulation of the biophysics of the adult human brain is certainly an interesting scientific endeavor, although it might be close to impossible, with the investigating tools we have today, but it is at the same time too much and too little for figuring out a feasible approach for the design of autonomous robotic agents that can be integrated usefully in the social and industrial environment of the 21st century: for convenience we may use the acronym COCOBOT, to refer to the dual requirements of CO-operation (with humans) and COgnition (which includes autonomous and prospection capabilities). Robots of this kind do not need to be world masters of chess or go and are not supposed to be fluent in any human language, although some rudimentary logical capacity (for autonomy) and some linguistic competence (for communication/interaction with human partners) is necessary. As a matter of fact, COBOTs or Collaborative robots are already in the market since more than a decade: they are designed to work in the vicinity of human workers, allowing close interaction with humans, without protective barriers, in a number of typical industrial applications, with the strict requirements of reactive-safety blocking the movements in case of unexpected collisions. Existing COBOTS can be programmed for specific tasks as pick&place, machine tool tending, packaging, assembly, surface finish, welding, dispensing of sealants, etc. However, they are designed to be efficient and not to be brain-inspired in any sense, lacking any degree of cognitive capability. On the other hand, the issue of safety is important and should be intrinsic also in the design of COCOBOTS; if applied to interaction with non-expert and/or fragile humans, safety can be achieved only in systems with cognitive abilities based on prospection. Safety is not limited to the *Bodyware* of COCOBOTS, involving a set of fast reactive mechanisms but is supposed to refer to proactive behaviors, real as well as mental, that identify the *Cogniware* of COCOBOTS.

In the previous sections of this paper we propose two pillars for the design of such Cogniware: (1) it should be embodied and (2) it should support adaptive behavior through dynamic lifelong processes starting from the analogy with a developmental process. This means, in particular, that Bodyware and Cogniware should be designed as a unitary organism, not two independent entities to be selected off the shelf and matched for any specific application. In particular, the developmental pillar suggests that a main challenge for the future research will be to identify a minimal embryonic structure, from which it is possible to bootstrap a developmental process of a prospection grounded system capable of action execution and simulation. Since we are still far away from any accepted and/or credible solution, it may be inspiring to listen to an imaginative novelist (Calvino, 1988) when he defines an “imagination machine” as follows: “…a kind of electronic machine that keeps track of all possible combinations and selects those that suit a particular purpose, or simply those that are the most interesting or pleasing or amusing.” This simple definition emphasizes the role of causality, purposiveness and the existence of a value system. Calvino’s “electronic machine” might be in the form of a minimalistic cognitive architecture with the components needed for prospection to emerge and that, in spite of its simplicity, could trigger complex behaviors. However, starting from such initial implementation, the critical issue remains how to outline the evolution process in a self-organizing manner.

Moreover, it is worth stressing that the embodiment pillar refers to both the Bodyware and Cogniware of such embryonic agents stressing the importance of the relationship between structure and function in the design of cogniware and the potentials offered by neuromorphic hardware/wetware that, even if still does not exist, is an active research area. For example, organic computers which, unlike conventional digital computers using binary states, are based on neural tissue (Vacher et al., 2008; Bray, 2009; Cai et al., 2023) or polymeric material (Krauhausen et al., 2021; Sarkar et al., 2022) capable of instantiating thousands of states and communicating with each other in a self-organized way, constantly forming and reforming new connections. In principle, such technologies might provide substantially more energy-efficient computing and this feature is in sharp contrast with the notorious energy voracity of AI technologies. Moreover, for organic computing technologies the classical distinction between hardware and software of the von Neumann computing architecture is supposed to disappear because the nature of computation is not Boolean algebra on strings of bits but the dynamic modulation of connection and disconnection of “organic neural assemblies” as an effect of the interaction of the organic brain with the environment through an organic body. This should evolve as a self-organizing process driven by Hebbian learning, at the microscopic level, and reinforcement learning, at the behavioral level. It is also expected that such self-organizing process could lead to the formation of self-organized maps (Kohonen, 1982) as well as topology representing networks (Martinetz and Schulten, 1994) that might be the building blocks for the maturation of the body schema throughout development.

At the same time, the same organic technologies that are being investigated for organic computing are also being considered for the development of neuromorphic sensors (Blaiszik et al., 2010; Hassan et al., 2015; Richardson and Cheneler, 2019; Krauhausen et al., 2021; Sarkar et al., 2022). Neuromorphic actuators or artificial muscles are also being investigated using a variety of technologies, such as semicrystalline polymer fibers, ionic-polymer/metal composites, twisted nanofibers, conducting polymers, etc., (Mirvakili and Hunter, 2018) and probably the challenge is greater in this case than for neuromorphic sensors. In summary, we suggested that COCOBOTs might be the target for establishing Artificial Cognition as the crucial approach for developing the Next-Generation of Autonomous Robotic Agents rather than one of the many forms of Artificial Intelligence applications. Furthermore, we suggest that the evolution of COCOBOTs is a work in progress that requires a multidisciplinary converging approach, both in the scientific and technological sense, in order to establish Artificial Cognition as the crucial approach for developing the Next-Generation of Autonomous Robotic Agents that can fit the requirements of 5IR, namely “the harmonious human–machine collaborations, with a specific focus on the well-being of the multiple stakeholders” (Noble et al., 2022).

## Data availability statement

The original contributions presented in this study are included in this article/supplementary material, further inquiries can be directed to the corresponding author.

## Author contributions

GS: Conceptualization, Formal Analysis, Methodology, Writing – review & editing. AS: Conceptualization, Formal Analysis, Methodology, Writing – review & editing. PM: Conceptualization, Formal Analysis, Methodology, Writing – original draft.

## Footnotes

1 To our knowledge the two papers (Taylor and Taylor, 2021; Iglesias, 2022) are the first ones to contain the term Artificial Cognition in the title.

2 Z.W. Pylyshyn: “if a system is cognitively penetrable then the function it computes is sensitive, in a semantically coherent way, to the organism’s goals and beliefs.”

3 This issue is related to the baseline architecture previously mentioned as the blueprint of evolution providing the scaffolding for innate behaviors (Zador, 2019), such as the observed prenatal synergies possibly facilitated by the viscous amniotic liquid.
